# Riding into Health: A Case Study on an Equine-Assisted Childhood Obesity Intervention

**DOI:** 10.3390/ijerph16234835

**Published:** 2019-12-01

**Authors:** Katy Schroeder, Jason Van Allen, Emily Dhurandhar, Brittany Lancaster, Zohal Heidari, Kandis Cazenave, Dianna Boone, Phyllis Erdman

**Affiliations:** 1 Department of Animal and Food Sciences, College of Agricultural and Natural Resources, Texas Tech University, Lubbock, TX 79409, USA; kandis.cazenave@ttu.edu; 2 Department of Psychological Sciences, College of Arts and Sciences, Texas Tech University; Lubbock, TX 79409, USAbrittany.lancaster@ttu.edu (B.L.); zohal.heidari@ttu.edu (Z.H.); dianna.boone@ttu.edu (D.B.);; 3 Department of Kinesiology and Sports Management, College of Arts and Sciences, Texas Tech University; Lubbock, TX 79409, USA; emily.dhurandhar@ttu.edu; 4 Department of Kinesiology and Educational Psychology, College of Education, Washington State University; Pullman, WA 99164, USA; perdman@wsu.edu

**Keywords:** obesity, children, equine-assisted interventions

## Abstract

In this article, we present an exploratory case study that describes the initial outcomes of the Equine-Assisted Positively Fit (EAPF) program. Children with obesity and their caregivers were recruited to participate in the eight-session program. Results indicated that treatment completers (*n* = 2) had a decrease in fat mass and fat mass percentage and an increase in fat-free mass and fat-free mass percentage. Moreover, results from accelerometer measurements of physical activity indicated that participants increased their moderate to vigorous physical activity, as well as reported increased self-efficacy for physical activity. Qualitative data from the post-intervention focus group suggested children perceived the treatment acceptable and enjoyable. Findings from this study provide support for future investigations on the feasibility and potential efficacy of pairing children and their caregivers with horses to accomplish health-related goals.

## 1. Introduction

Obesity, also defined as excess body fat, is a complex disease affecting over a third of the world’s population [1]. The scope of the disease is significant, especially for young people. Childhood obesity represents the early stages of this chronic disease and can lead to early onset of medical comorbidities such as high blood pressure, and increased metabolic and cardiovascular risk [2]. According to current estimates in the United States, one in five school-age children and adolescents has obesity [3], and access to evidence-based childhood obesity care is extremely low compared with the need for treatment of this disease [4]. Psycho-social and behavioral comorbidities associated with childhood obesity include internalizing disorders (e.g., anxiety or depression), low quality of life, sleep problems, body dissatisfaction, eating disorders, and bullying from peers [5,6,7,8].

It has been recommended that obesity treatments for youth should focus on environmental factors such as dietary and physical activity behaviors that are modifiable [9], rather than focusing primarily on overweight and weight control. The latter focus could be demoralizing to young people and lead to poor body image, fear of food, self-consciousness around peers, negative beliefs about physical abilities, and low self-worth [10]. Consequently, research indicates that treatment should consist of multicomponent models that are grounded in positive health education and delivered by a multidisciplinary team [4,10,11]. Successful weight loss is also more likely to occur when individuals actively engage in the behaviors known to reduce weight [12]. For example, youth report a preference for exercising to improve their health and value peer support formats that could be fun, as well as educational [13]. Additionally, children’s sense of hope [14] and feelings of self-efficacy may influence their readiness to initiate positive health behaviors [15].

Empirical studies also demonstrate that youth may benefit the most from interventions that combine cognitive behavioral components with parental participation, physical activity, and dietary components [16]. For instance, youth (*N* = 93) who participated in a family-based, group intervention called Positively Fit experienced significant reductions in body mass index (BMI) and BMI percentile, as well as significant increases in self-reported quality of life [17].

Novel approaches to childhood obesity interventions hold the promise of making these services more engaging and attractive to children and families. Given the growing evidence base for integrative obesity prevention programs [18], as well as youth treatment preferences, innovative approaches such as equine-assisted activities and therapies (EAAT) may have an additive effect on youth health outcomes. Indeed, a recent review of integrative treatments for pediatric obesity suggested that animal-assisted therapies have potential to help children [19]. Specifically, Boisvert and Harell suggested that connecting with an animal companion leads to more feelings of joy, hopefulness, self-confidence, motivation, and commitment to engage in positive health behaviors. Utz and colleagues [20] reported that various types of human–companion animal interactions, such as dog walking, can have positive influences on child and adolescent health; however, less is understood about how this process occurs. Additionally, previous studies have produced mixed results. For instance, it remains uncertain if activities like dog walking improve physical health, or if people bring dogs into their homes because caring for a dog would match their already active lifestyles [20]. Accordingly, it is important to continue exploring how and why different types of human–animal relationships affect children’s health-related behaviors. Relatedly, few studies exist on incorporating animals in clinical settings for the treatment of childhood obesity, which necessitates further inquiry.

Increasing empirical evidence suggests that EAATs positively influence child and adolescent health. These findings also align with physical, psychological, and behavioral treatment targets for childhood obesity mentioned earlier in our review of the literature. For example, previous studies indicate that youth participating in EAAT interventions experience gains in social support [21], less anxiety and depression [22,23], and improved self-concept [24]. Literature also affirms that EAAT interventions may increase young people’s persistence during difficult tasks [21], support life skill development [23,25], and facilitate client–mental healthcare provider rapport [26]. In empirical investigations focused specifically on therapeutic riding [27], or horsemanship skills with a riding component [24], children and teens have reported experiencing a better quality of life and more self-competence [27], as well as more self-confidence, self-efficacy, and self-mastery [24]. To date, only one study has explored the effects of equine-assisted activities on childhood weight management [28]; however, no research exists on integrating human–equine interactions into a standardized, evidenced-based childhood obesity intervention. Therefore, the primary objective of this investigation was to explore child outcomes resulting from the addition of equine-assisted activities to an already standardized curriculum for the treatment of childhood obesity.

## 2. Materials and Methods

### 2.1. Study Design

We conducted a mixed-method approach, which entailed a one-group, pre–post intervention study, followed by a focus group, to evaluate our primary physiological and psychological outcomes of interest, as well as to gain an understanding of the intervention as it was experienced by the participants. The study was undertaken by an interdisciplinary team of researchers in Psychological, Kinesiology, and Animal Sciences. We collected data for this study between January 2018 and June 2018.

### 2.2. Participant Recruitment

Prior to recruitment, we obtained Institutional Review Board (IRB2017-628) and Institutional Animal Care and Use Committee (17052-12) approvals. We invited children with current overweight or obesity, ages 9–12 years old, and their primary caregivers, to participate in the study. Participants were recruited from a waitlist at a university-based psychology clinic. Due to group size restrictions, 4 families from the waitlist were queried about their interest in participation, while 5 children on the clinic waitlist were automatically excluded because they did not meet the age range criteria. One family that indicated interest later declined to attend the informed consent meeting due to family issues and being unable to commit to sessions on the scheduled day and time. In order to ensure the safety of family groups working with therapy horses, individual members of family groups were excluded from study participation if they experienced breathing difficulties or allergic reactions to the environment in which a respiratory emergency was likely to occur, had mobility issues that would prevent them from comfortably walking without assistance, or were pregnant. Exclusion of one member of the child participant’s family did not exclude the entire family group, only that particular member. Additionally, children were excluded from study participation if their weight was more than 100% above the median BMI for age and gender; if either the child or their primary caregiver did not speak English; if a child was diagnosed with medical conditions known to promote obesity; or if a child was enrolled in another weight control program and/or taking weight-affecting medications. Lastly, children weighing more than 220 pounds were excluded from study participation due to weight-carry restrictions of the therapy horses. Prescreening phone interviews were conducted by a member of the study team. Children and their caregivers who met the initial eligibility criteria and confirmed availability to attend the weekly interventions sessions were then invited to an informed consent meeting, which was immediately followed by the collection of baseline measurements. Three children met the initial screening criteria and were enrolled in the study.

### 2.3. Intervention

Table 1 shows an outline of the Equine-Assisted Positively Fit (EAPF) program, which is an adaptation of the Positively Fit curriculum [29], an evidenced-based, 10 week, manualized intervention that covers topics related to eating habits/dietary choices, increasing exercise, and helping parents navigate the challenges of making changes in their family to support their children’s lifestyle efforts. A team of clinical psychology graduate students supervised by a Ph.D.-level clinical supervisor, and a counselor certified as a Professional Association for Therapeutic Horsemanship, International (PATH, Intl.) Equine-Specialist in Mental Health and Learning (ESMHL), facilitated the group. The program involved eight, two-hour sessions held at a PATH International Premier Accredited EAAT center in the southwestern United States. When designing this program, we took into account recommendations from the literature suggesting mixed-format approaches (i.e., some time with individual families and some group time) could result in better treatment effects than group-only approaches [4]. Thus, we paired each child and their caregivers with an individual horse. This allowed us to tailor the delivery of content for each family’s particular goals. In addition, we assigned a trained horse handler to each family to assist with safety and skill development of the participants, as well as to monitor the welfare of the therapy horses. The general format involved group-as-a-whole discussions and delivery of psychoeducational content to caregivers and children separately before and after an equine-focused activity. The equine-assisted activities involved horse care, handling, and horseback riding, and each activity was linked to content from the Positively Fit curriculum.

### 2.4. Procedures

Upon confirmation of eligibility, youth and their caregivers were brought in for an initial appointment six days before the start of the intervention. During this time, caregivers completed informed consents and youth signed assents. Additionally, they completed intake paperwork for the EAAT facility, answered questionnaires, and had anthropometric measurements taken. A research assistant was available at all times to answer any questions regarding consents or questionnaires. During this visit, youth were given an Actigraph GT3X+ accelerometer to wear for six days prior to the start of the intervention. The youth were instructed to wear the Actigraph GT3X+ on their nondominant wrist 24 h a day. Youth and their caregivers then attended 8 weekly sessions at the EAAT center. At the end of each session, parents and children were administered the Group Session Rating Scale/Child Group Session Rating Scale and given a Critical Incident Questionnaire to complete at home, then return to the research team at the next session. The final Critical Incident Questionnaires were returned during the post-intervention data collection visit. After the last session, a post-intervention data collection was scheduled within a month to re-administer questionnaires, take anthropometric measurements, and give the child an Actigraph GT3X+ accelerometer to wear for 7 days after the intervention concluded. After the post-intervention data collection, children and their caregivers attended a focus group.

### 2.5. Measures

#### 2.5.1. Body Composition

Body composition was measured with the Imp SFB7 (ImpediMed Limited, Brisbane, Australia). This instrument uses bioelectrical impedance spectroscopy analysis, which passes an electrical current through the body and based on the resistance to the current can determine fat mass, muscle mass, and body fat percentage. The Imp SFB7 is a research-grade, tetrapolar instrument (i.e., hand-to-foot, whole body technique) that provides a high level of accuracy for body composition measurement. Typically, the measurement is performed in fasted individuals, but taking the measurement fasted was not possible for this study. However, the pre- and post-measurements were both taken in the late afternoon, around the same time of day to minimize time-of-day variation.

#### 2.5.2. Physical Activity

The Actigraph wGT3X+ measures physical activity in three orthogonal planes (vertical plane, antero-posterior, and medio-lateral) and provides a composite vector magnitude of these three axes (VM3; [29]). The Actigraph wGT3X+ has been shown to be a valid measure of physical activity [30]. A sampling rate of 60 Hz was used, and accelerometer data was measured in 60s epochs.

#### 2.5.3. Self-Efficacy for Health Eating and Physical Activity (SE-HEPA)

The SE-HEPA [15] is a self-report measure consisting of two, eight-item scales that measure children’s self-efficacy for healthy eating and physical activity. Each item is rated by the child on a 5-point scale from “disagree a lot” to “agree a lot”. In a pre-adolescent sample, the instrument demonstrates good reliability (αs > 70) and correlational analyses suggest a significant relationship between the two factors (i.e., healthy eating, physical activity) and preadolescent body mass index [29].

#### 2.5.4. Pediatric Quality of Life Inventory (PedsQL)

The PedsQL Generic Core Scale is a 23-item self-report measure of youth quality of life [31]. Items encompass four dimensions including physical (8 items), emotional (5 items), social (5 items), and school (5 items) functioning. The PedsQL self-report has demonstrated good internal consistency.

#### 2.5.5. Children’s Depression Inventory Self-Report (Short Version) (CDI 2:SR(S))

The CDI 2:SR(S) Form is a 12-item, self-report screening measure of depressive symptoms. The CDI 2:SR(S) has acceptable psychometric properties and yields a Total Score that is generally comparable to the one produced by the full-length version [32].

#### 2.5.6. Child and Adolescent Scales of Hope (CASH)

The CASH [33] is a 9-item self-report measure of child and adolescent hope and is a revision of the original Children’s Hope Scale [34]. The CASH evaluates all three subcomponents of hope including Goals (3 items), Agency (3 items), and Pathways (3 items). Item responses are measured on a 6-point scale, in which the participant reports the extent to which a statement describes them “none of the time” to “all of the time.” Higher scores on the CASH indicate higher levels of hope. A confirmatory factor analysis of the 3-factor structure of the CASH demonstrated good fit [33]. The CASH has demonstrated high reliability (ρ = 0.92) and a moderate test–retest reliability (r = 0.59, *p* < 0.01) [33].

#### 2.5.7. Child Group Session Rating Scale (CGSRS)

The CGSRS [35] measures group therapy alliance for children ages 6–12. It is a 4-item visual analog measure, in which the child is instructed to place a mark on each 10 cm line. A mark placed further to the left indicates more negative feelings, and a mark placed further to the right indicates more positive feelings. Items address four areas related to the child’s group session experience: “relationship” (“The leader and group listened to me”), “goals and topics” (“We talked about and did important things”), “acceptability” (“I liked what we did today”), and “overall fit” (“Today was good for me, I felt like a part of the group”). CGSRS scores are calculated by measuring the marks made by the child and summing each length to the nearest centimeter. The four scores are summed for a possible score of 40.

#### 2.5.8. Critical Incident Questionnaire (CIQ)

The CIQ [36] was developed to capture group members’ most meaningful moments in group therapy. A modified version of the CIQ was used to query child and adult participants about what they considered to be the most important event in each group session. The CIQ consists of one question (“What was the most important event for you that happened in the last group session?”) with sub-prompts asking participants to describe what happened during the group, who was a part of the event, why the event was important, and what learning took place. The wording of prompts in the questionnaire were modified to be developmentally appropriate for child participants.

#### 2.5.9. Parent Efficacy for Child Healthy Weight Behavior Scale (PECHWB)

The PECHWB [37] is a 41-item self-report measure assessing parent self-efficacy for children’s weight behaviors across four subscales: minimizing fat and sugar intake, reducing sedentary activities, eating recommended servings of fruit and vegetables, and physical activity. Parents rate their confidence to support a child across the four weight reduction barriers on a scale of 0–100 (0 = “not at all confident” to 100 = “extremely confident”). The PECHWB full scale and subscales demonstrate high internal consistency (α > 90) and good convergent validity, as evidenced by significant positive correlations with a general measure of parenting self-efficacy [37].

#### 2.5.10. Group Session Rating Scale (GSRS)

The GSRS [38] is a group therapy alliance measure for adults, which consists of a 4-item visual analog scale. Respondents are instructed to a place a mark on each 10 cm line. A mark placed further to the left indicates more negative feelings, and a mark placed further to the right indicates more positive feelings. Items cover four areas related to a group session experience: “relationship” (“I felt understood, respected, and accepted by the leader and the group”), “goals and topics” (“We worked on and talked about what I wanted to work on and talk about”), “acceptability” (“The leader and group’s approach is a good fit for me”), and “overall fit” (“Overall, today’s group was right for me—I felt like a part of the group”). GSRS scores are calculated by measuring the marks made by the respondent and summing each length to the nearest centimeter. The four scores are summed for a possible score of 40.

#### 2.5.11. Focus Group Interview

Caregivers and children participated in a one-hour, semi-structured focus group discussion regarding the following topics: group format, length, session content, group activities, working with horses, experiences with the facilitation team, any challenges or successes experienced during the program, and any additional feedback participants wanted to share about their experiences. The purpose of this focus group was to further determine the effects of the EAPF program on participants and guide refinement of the curriculum. The focus group was moderated by a graduate student member of the research team. The meeting was audio-recorded, and participants’ feedback was transcribed verbatim into electronic format.

### 2.6. Data Analyses

Due to the limited sample size, no inferential statistical analyses were conducted. Instead, composites scores for measures were created, and pre- to post-intervention change scores were utilized by subtracting the pre-test scores from post-test scores. With regard to the physical activity data, ActiLife software version 6.13.4 was used to analyze Actigraph GT3X+ data. The physical activity data provided by the accelerometer includes time spent in sedentary, light, moderate, and vigorous physical activity data.

Qualitative data from the focus group interview was analyzed using inductive qualitative content analysis [39]. First, the transcribed interview document was downloaded into Nvivo 12 Plus (QRS International, Melbourne, Australia), a qualitative analysis software package, by a member of the research team. The researcher prepared for data interpretation by reading the transcript to familiarize herself with the raw data, then read it again, line-by-line to identify and label meaning units (i.e., participant perspectives). Open coding of each meaning unit, analytic memoing, and constant comparison were utilized in a recursive process to identify and describe emerging concepts from participant statements, then sort and group similar codes together, followed by abstraction of sub-categories and the main categories. Once the main categories and category descriptions were established, representative quotations were linked to each category. To increase trustworthiness, a second member of the research team, not involved with intervention delivery, independently reviewed the categories, category descriptions, and representative participant quotations. The two researchers discussed discrepant viewpoints and revised results from the coding and categorization process until consensus was achieved.

## 3. Results

Two out of the three child participants completed treatment, which we defined as attending two-thirds or more of the treatment sessions. Child 1 attended eight out of eight sessions, and Child 2 attended six out of eight sessions. Child 3 attended two sessions before dropping out of the study due to circumstances unrelated to his involvement in the study. Thus, we considered Child 3 a treatment non-completer, and he was not included in our data analyses.

### 3.1. Quanitative Results

#### 3.1.1. Child 1 Outcomes

Child 1’s primary outcomes are displayed in Table 2. Child 1 was a 12-year-old male with a baseline weight of 97 kg, baseline height of 164.6 cm, and a BMI of 36. He experienced a 21 min average increase of moderate to vigorous physical activity (PA) per day from pre- to post-assessment and gained 1.5% fat-free mass while losing 1.5% body fat. His BMI and BMI percentile remained stable over the course of the intervention. Child 1 did not experience an increase in healthy eating self-efficacy, but did experience an increase of 7 units in physical activity self-efficacy. Additionally, Child 1 experienced increases in hope (∆ = 18, baseline hope = 28, post hope = 46) and quality of life (∆ = 42.39, baseline quality of life = 46.74, post quality of life = 89.13). Child 1’s total depressive score at post-treatment could not be computed due to missing data (baseline CDI 2: SR(S) T-score = 88). This baseline score indicates that Child 1 was experiencing very elevated depressive symptoms. Overall, he reported a very positive experience in the group with an average rating of 40 on the CGSRS (eight administrations).

#### 3.1.2. Child 2 Outcomes

Child 2’s primary outcomes are displayed in Table 3. Child 2 was a 10-year-old male with a baseline weight of 61.9 kg, baseline height of 144.4 cm, and a BMI of 29.7. He experienced a 17 min average increase of moderate to vigorous PA per day from pre- to post-assessment and gained 2% fat-free mass while losing 2.1% body fat. His BMI and BMI percentile remained stable over the course of the intervention. Furthermore, both his self-efficacy for healthy eating and physical activity increased over the intervention. Child 2’s total hope scores could not be calculated due to missing data; however, his agency subscale score and pathways subscale score could be compared (∆ = −2, baseline agency = 13, post agency = 11; ∆ = −1, baseline pathways = 9, post pathways = 8). These differences are unlikely to be clinically significant. Child 2 experienced no meaningful changes in depressive symptoms (∆ = 0, baseline CDI 2: SR(S) T-score = 57, post CDI 2: SR(S) T-score = 57) or quality of life (∆ = −7.34, baseline quality of life = 51.09, post quality of life = 41.75). He had a mean total score of 40 on the CGSRS (six administrations).

#### 3.1.3. Caregiver Outcomes

Caregiver scores for the PECHWB were available pre and post for one of Child 2’s caregivers. The caregiver’s total average score on the scale increased from 33 to 56 over the course of the intervention. For Child 1, a different caregiver attended the post-intervention data collection meeting. Thus, we were unable to collect a post-measurement of the PECHWB from the caregiver who attended the pre-intervention data collection meeting. The caregivers who completed the GSRS evidenced strong positive feelings about the group experience; one of Child 1’s caregivers completed seven administrations of the GSRS for a mean total score of 39, and a second caregiver for Child 1 had a total score of 40 on the GSRS for one administration completed. One of the caregivers for Child 2 completed five administrations of the GSRS for a mean total score of 40, and the second caregiver for Child 2 had an average total score of 40 across three administrations completed.

### 3.2. Qualitative Results

Due to a combination of low compliance and readability issues with hand-written responses to the CIQ questionnaires returned to the research team, we were unable to conduct a qualitative analysis of data from this instrument.

#### Focus Group Interview

Each child that completed the EAPF program was accompanied by one of their caregivers, which resulted in a total of four focus group participants. Participants’ responses to the focus group questions yielded four major categories: (1) equine contributions to participant experiences, (2) group climate, (3) learning outcomes, and (4) improving the group experience.

• Category One: Equine Contributions to Participant Experiences

Both child and caregiver participants described the inclusion of horses as a motivating factor to attend treatment sessions. One caregiver reported that coming to sessions was “a reward” for her child, and the other caregiver described the presence of horses as “something [for him] to look forward to” after school. Caregivers also indicated that their children developed feelings of affinity toward the horses. One caregiver reported that she expected her child to be nervous around horses, “So the way he took to [the horse] was kind of surprising to me.” She also noted that after the first session, her child would excitedly tell her, “Don’t forget Friday; don’t forget Friday!”, which further illuminated the child’s interest in seeing his horse again.

Specific horse–human interactions also appeared to contribute to the children and caregivers’ enjoyment of the treatment sessions. Both children stated that they liked the horseback riding sessions. In particular, one child elaborated that his favorite equine-focused activity was learning to ride his horse through an obstacle course. The other child participant mentioned that he also liked leading his horse. The caregivers reported that grooming horses with their children was a fun activity. One caregiver stated that she liked how the grooming activity provided a special opportunity for her to bond with her son, “I liked that connection, so umm; I like any connection I can get with him.” The family horse grooming activity appeared to be a particularly salient experience for one of the child participants. He spoke up after his caregiver reported that grooming “was fun” to mention that he got to show one of his caregivers how to clean the horse’s hooves. This statement suggested to us that the child may have felt a sense of pride about his ability to master this skill and teach it to his caregiver.

• Category Two: Group Climate

The category of group climate encompassed aspects of the multi-family group therapy format that fostered a positive learning environment reflective of care and support among group members and the group leaders. The caregivers and one child participant voiced a preference for the multi-family group format, while the other child participant did not verbalize a preference. Both caregivers reported that the group format allowed them to share advice with each other regarding strategies for healthy eating and exercise. A caregiver speaking on behalf of her partner, who was absent from the interview, stated that, “he liked the discussions, and everybody talking, getting other family information, and trying to use that helpful information on both sides.” Participants also described how the program facilitators contributed to the positive group climate. For example, when asked about what it was like to work with the facilitators, one caregiver said, “It was awesome. I liked it… they made you feel comfortable and if you didn’t understand something, they’d help you understand it better.” On a similar vein, one of the child participants mentioned that the facilitators were “good listeners” and “they did a lot of good things for us, they’re kind, helpful…” Lastly, evidence of a positive group climate was found in a caregiver’s comment that, “We enjoyed it. It gave [my child] something to look forward to on Fridays, and [gave] him a goal-you know, to work for, and make the right choices.”

• Category Three: Learning Outcomes

The category, learning outcomes, reflected the ways in which curriculum topics and equine-assisted activities contributed to children’s lifestyle changes and the successes and challenges that families experienced when applying the curriculum content at home. Evidence of participants’ learning outcomes were derived from their explicit statements and other noteworthy responses to moderator questions. For example, when asked about ways to improve the group experience, a child participant suggested we include a “yellow food” snack sometimes, and the other child suggested “apples” as the specific snack food to include. The children’s input clearly demonstrated that that they understood the Traffic Light System, which was a topic designed to help children make healthier food choices. The salience of this learning outcome, and the helpfulness of this particular topic, was supported by one parent sharing that “we talk about the traffic light all the time” and her report that her child had success in choosing healthy foods:

Like when we go out to eat, cause we’re all very busy, so we eat out a lot. So he’s started making better decisions. Like, he would have a salad, or just a, not a double meat hamburger, you know, like he would start making conscious decisions, uh, vegetables instead of fries, so, but he did it on his own, like I wouldn’t have to remind him.

In response to this sharing, the other caregiver followed up with a statement that suggested her child also had learned how to use the Traffic Light System to monitor his eating habits. She noted that, “Yeah, I’d have to say [my son] noticed it too, like, ‘I can’t have that.’”

Regarding topics participants mentioned as most helpful, one caregiver initially reflected that all the topics were “good” and in particular, she thought the topic on emotions and eating was helpful, implying that boredom may sometimes contribute to her child’s eating habits, “We notice his increase of eating, and it’s more than what it is when we have a busy day.” One child voiced agreement that the discussions about the Traffic Light System and emotions and eating were “good”, and he also found the content on bullying solutions to be a helpful topic. Participants also described specific equine-assisted activities that supported their learning process. Specifically, learning to redirect the horses’ attention away from eating grass during a leading activity helped one child learn about stimulus control. In addition, the other child’s caregiver explained that learning to lead the horses taught her child patience, which she felt was related to sticking with lifestyle changes. She linked developing patience to a particular challenge she experienced with her child regarding his follow-through with portion control. Lastly, both caregivers agreed that practicing the skill of praise and reward during one of the children’s horseback riding sessions was a helpful activity. One caregiver stated, “Oh yeah, the praise part was awesome. Doing the praise worked really good, so, you could see it in their faces that it made their day and stuff…”

• Category Four: Improving the Group Experience

The last major category contained participants’ feedback for improving the group experience. Overall, participant feedback was focused upon logistical considerations, the group topics, and the equine activities. Both caregivers and one child voiced a preference for expanding the 8 week program to a 10 week program. Additionally, a caregiver and one of the children reported the two-hour sessions were good, and the other child said he would prefer one-hour sessions. However, he did not elaborate about his reasons for wanting the sessions to be shorter. The topic of fitness education became a focal point for discussion during the focus group. One child and both caregivers shared that they would like to learn more information about fitness, including types of fitness activities and how to make a fitness schedule. They also expressed a desire to do a family fitness activity with the horses. One of the children and his caregiver also expressed that they wanted more time with the horses during the program. Finally, one caregiver suggested it might be helpful in the future for the curriculum to include information specifically about the negative health consequences associated with unhealthy eating and sedentary behavior.

## 4. Limitations

The primary limitations of this exploratory study involve the small sample size, geographic location (i.e., rural setting in the southwest United States), as well as a lack of comparison or control group. Therefore, the effects of this intervention cannot be generalized to other children experiencing obesity, nor can we conclude which components of this multicomponent model had the greatest impact on child outcomes. Future studies are needed to isolate the effects of equine-assistance as compared with treatments without equine involvement, as well as replication studies to determine if positive results can be achieved when certain aspects of the intervention change, such as the location, facilitation team, and horses involved. Lastly, another limitation of the small sample size and the research methodology is that we were unable to make conclusions regarding the feasibility of executing a larger experimental trial; thus, some elements required to test feasibility, such as participant acceptance of randomization procedures and calculation of recruitment rate [40], were not included in the study design.

## 5. Discussion

Overall, our quantitative and qualitative findings indicate that the intervention may have contributed to positive impacts on the health and physical functioning of the child participants. An important finding from this study was that each child’s BMI and BMI percentile remained stable. This is a positive accomplishment given expert recommendations for weight loss or weight maintenance during linear growth for youth [9]. Body composition also improved for both children, with an increase in lean mass and decrease in fat mass. Furthermore, both children increased their moderate to vigorous physical activity across the intervention, and physical activity self-efficacy increased for both children.

Self-efficacy for physical activity tends to be particularly low in children with obesity and may pose a barrier to participating in physical activity [41]. Given that physical activity is a critical part of childhood obesity management, any intervention that improves physical activity, and helps children develop self-efficacy for physical activity, may be advantageous. Children in this study evidenced increased physical activity self-efficacy; however, this preliminary study does not allow us to make solid conclusions regarding the degree to which the equine activities contributed to this particular improvement, nor if the equine-assisted activities had a direct influence on children’s MVPA (average minutes of moderate-to-vigorous physical activity per day). Indeed, Self-Efficacy for Physical Activity (SEPA) subscale scores could have improved due to other factors, such as caregiver encouragement and behavioral reinforcement. Furthermore, qualitative data from the focus group interview led us to question if the child participants were truly aware of how much physical activity they achieved while working with the horses, especially since one child stated a suggestion to add fitness activities with the horses. Consequently, it may be important to incorporate more explicit information at the beginning of the group experience regarding how the equine-assisted activities contribute to increasing PA, and future research should explore if equine-assisted activities have an independent effect on physical activity self-efficacy in childhood obesity.

Well-being of the children may have also improved with this intervention. Child 1 experienced increases in quality of life and hope. Although Child 2 did not experiences increases in quality of life or hope, he also did not experience decreases in these areas, which would have likely been clinically meaningful. These findings therefore indicate that hypotheses proposed by Boisvert and Harell [19] about animal-assisted activities leading to positive behavior change through hopefulness and joy may be plausible; however, previous studies on non-equine weight management programs have also shown increases in quality of life and self-esteem and decreases in depressive symptoms [42,43]. Therefore, a natural next step in researching this program will be to compare the results from an equine-assisted group with the traditional clinic program that does not include equine interactions.

Data from one family also would suggest that parental confidence in managing their child’s weight improved with the program. Recent studies suggest that parental ability to manage child weight is actually equally or more important than the child’s skills, as interventions that focus on parents alone are quite effective [44]. Therefore, future studies could explore the relative pros and cons of interventions that focus on parents alone and those that focus on the whole family in an equine-assisted setting.

Attendance in pediatric obesity treatment, including family-based interventions, is considered to be an ongoing challenge [45,46]. Risk factors contributing to treatment noncompletion include male gender, depressive symptomology, as well as family dysfunction. Two out of three child participants completed the EAPF intervention. This was encouraging to our research team given attrition issues in childhood obesity treatment. The novel, equine-focused activities could have played an important role in children’s motivation to attend sessions, which was supported by our qualitative findings from the focus group interview. In particular, participants voiced enthusiasm for the equine activities and expressed a desire for more time with the horses. Furthermore, the caregivers’ motivation may have been impacted by the opportunity to interact with horses, possibly contributing to their commitment to attend sessions with their children. However, we cannot rule out that treatment completion also may have been a function of the group versus individual family format. For instance, previous studies indicate children appear more motivated to keep attending group-based interventions because of the opportunities to engage in fun, hands-on activities while making new friends with peers who have similar levels of activity and weight status [47].

Regarding study instrumentation, we observed low compliance with completion of the take-home, open-ended questionnaire (CIQ), which suggested to us that future studies might garner better data through real-time, qualitative assessments of participant experiences, to include video or audio recordings, rather than asking participants to retrospectively recall how they felt, and then remember to bring documentation to the next session. This would also have a tangible benefit of reducing overall study participation burden for both children and caregivers.

## 6. Conclusions

The purpose of this study was to conduct a mixed-method exploration of adding equine-assisted activities to a standardized curriculum for childhood obesity treatment. Importantly, our results provide a pathway to design clinical trials of the EAPF program in the future. In particular, study participants found the treatment and its duration agreeable, evidenced by gains in several areas linked to successful outcomes associated with childhood obesity treatment, and reported that the equine activities were helpful. Furthermore, participants voiced a desire for more sessions, which suggested that this approach could have a unique effect on child motivation. Lastly, we observed distinct effects from the group therapy environment itself, primarily associated with the positive peer-to-peer and group leader-to-member interactions. This warrants further research regarding how and when the inclusion of horses contributes in facilitative ways to a positive group climate. Finally, given that childhood obesity is a critical public health issue, we hope the description of this novel equine-assisted program will serve to jumpstart more research on how time with horses might improve the health and wellbeing of children experiencing obesity.

## Figures and Tables

**Table 1:** Overview of the Equine-Assisted Positively Fit curriculum.

Positively Fit Topics	Equine-Assisted Activities	Purpose of Equine-Assisted Activities
Session 1: Getting to Know You	Equine safety demonstration; horse grooming	Trust-building; establishing ground rules to work safely with horses; exploring parallels between caring for horses and caring for one’s own health; increasing supportive behaviors among family members
Session 2: Getting the Bad Foods Out: (Stimulus Control and Traffic Light System)	Horse handling skills: learning to lead horses across different surfaces	Caregiver–child communication, exploring concept of stimulus control by learning how to redirect the horses’ attention away from areas that present stronger distractions (e.g., walking across grass)
Session 3: Praise and Reward for Healthy Lifestyle Choices	Children’s horseback riding lesson	Reinforce the concepts of praise and reward. Children learn how to praise themselves and their horses for accomplishments during the riding lesson; caregivers practice giving specific praise to their children
Session 4: Modeling Health Lifestyle Choices; Family as a Team	Equine observation activity; horse handling skills: learning to lead horses through an obstacle course	Families observe how horses explore novel objects in the arena and learn how to lead horses around obstacles in the arena. During observation, families are encouraged to notice how the horses differ in energy level, taking initiative to explore objects, and which horses follow or lead. Both activities help families become aware of their relational dynamics and communication styles that may support or hinder health lifestyle choices
Session 5: Emotions and Eating; Bully and Bullying Solutions	Children’s horseback riding lesson	Children learn to identify and share the emotions they experience while learning to ride horses through an obstacle course
Session 6: Physical Activity and Sedentary Behavior; Family Factors Influencing Weight	Children’s horseback riding lesson	Children learn how different breeds of horses need different types of exercise; Children learn how to take horses’ vital signs before and after the riding lesson to determine the horse’s level of fitness; children are instructed to work on keeping their horses active and reduce the amount of time the horses are sedentary during the riding lesson. This facilitates discussions around each child’s unique needs for physical activity and how to support them in reducing sedentary behaviors
Session 7: Problem-Solving with Stimulus Control	Children’s horseback riding lesson	Children practice riding their horses through a more challenging obstacle course with help from their caregivers, and then independently. Provides opportunities to discuss how children problem-solve and how caregivers can support problem solving during times it is more difficult to eat healthfully (e.g., special occasions)
Session 8: Looking Ahead	Horse grooming; horse leading game: red light/green light	Families spend quality time taking care of the horses they worked with, and then lead horses in a game of red light/yellow light/green light in which the *Traffic Light System* is reinforced

**Table 2:** Child 1 results.

Outcome Measure	Pre-Intervention	Post-Intervention
Height (cm)	164.63	167
Weight (kg)	97.63	100.3
BMI	35.96	36.04
BMI %	>99	>99
Fat Mass %	46.37	44.55
TBW % ^1^	39.26	40.59
Physical Activity (MVPA) ^2^	13	34
CASH	28	46
CDI 2: SR(S)	88	Missing Data
PedsQL	46.74	89.13
Healthy Eating Self-Efficacy	35	33
Physical Activity Self-Efficacy	18	25

^1^ TBW = Total Body Water; ^2^ MVPA = Average minutes of moderate-to-vigorous physical activity per day. BMI: body mass index; CASH: Child and Adolescent Scales of Hope; CDI 2: SR(S): Children’s Depression Inventory Self-Report (Short Version); PedsQL: Pediatric Quality of Life Inventory.

**Table 3:** Child 2 results.

Outcome Measure	Pre-Intervention	Post-Intervention
Height (cm)	144.40	145.5
Weight (kg)	61.96	63.3
BMI	29.9	29.76
BMI %	>99	>99
Fat Mass %	45.05	42.88
TBW % ^1^	40.22	41.81
Physical Activity (MVPA) ^2^	8	21
CASH	30	Missing Data
CDI 2: SR[S]	57	57
PedsQL	51.06	41.75
Healthy Eating Self-Efficacy	11	21
Physical Activity Self-Efficacy	23	34

^1^ TBW = Total Body Water; ^2^ MVPA = Average minutes of moderate-to-vigorous physical activity per day.
